# Injury and performance related biomechanical differences between recreational and collegiate runners

**DOI:** 10.3389/fspor.2023.1268292

**Published:** 2023-09-14

**Authors:** Ryan J. Evans, Tyler J. Moffit, Peter K. Mitchell, Derek N. Pamukoff

**Affiliations:** ^1^ School of Kinesiology, Western University, London ON, Canada; ^2^ Department of Kinesiology, California State University, Bakersfield, CA, United States; ^3^ Department of Kinesiology, California State University, Fullerton, CA, United States

**Keywords:** running, biomechanics, injury, performance, speed, gait, propulsion

## Abstract

**Introduction:**

Running related injuries (RRI) are common, but factors contributing to running performance and RRIs are not commonly compared between different types of runners.

**Methods:**

We compared running biomechanics previously linked to RRIs and performance between 27 recreational and 35 collegiate runners. Participants completed 5 overground running trials with their dominant limb striking a force plate, while outfitted with standardised footwear and 3-dimensional motion capture markers.

**Results:**

Post hoc comparisons revealed recreational runners had a larger vertical loading rate (194.5 vs. 111.5 BW/s, *p* < 0.001) and shank angle (6.80 vs. 2.09, *p* < 0.001) compared with the collegiate runners who demonstrated greater vertical impulse (0.349 vs. 0.233 BWs, *p* < 0.001), negative impulse (−0.022 vs. −0.013 BWs, *p* < 0.001), positive impulse (0.024 vs. 0.014 BWs, *p* < 0.001), and propulsive force (0.390 vs. 0.333 BW, *p* = 0.002). Adjusted for speed, collegiate runners demonstrated greater total support moment (TSM), plantar flexor moment, knee extensor moment, hip extensor moment, and had greater proportional plantar flexor moment contribution and less knee extensor moment contribution to the TSM compared with recreational runners. Unadjusted for speed, collegiate runners compared with recreational had greater TSM and plantar flexor moment but similar joint contributions to the TSM.

**Discussion:**

Greater ankle joint contribution may be more efficient and allow for greater capacity to increase speed. Improving plantarflexor function during running provides a strategy to improve running speed among recreational runners. Moreover, differences in joint kinetics and ground reaction force characteristics suggests that recreational and collegiate runners may experience different types of RRI.

## Introduction

Running is a popular form of physical activity with global participation (1). For some, running serves as a recreational activity, and others participate as competitive athletes. Various health benefits result from running including a reduced risk of cardiovascular disease, and overall healthier lifestyle (2). Despite the health benefits from participation, running has a high injury incidence rate (3, 4). For example, 37%–63% of those who participate in running suffer from some form of running-related injury (RRI) annually (3, 4). Some RRIs persist and could have lasting outcomes that impact runners' ability to participate, their health, and quality of life (5).

Most RRIs result from overuse and repetitive loading on the lower body (6, 7). Running is repetitive and involves cyclical mechanical loading applied to the lower extremity. Overuse of the lower limb structures in combination with potential predisposing risk factors contribute to the likelihood of injury incidence (7). Behavioural differences between recreational and competitive runners (e.g., quantity, frequency, intensity) also contribute to an increased risk of an RRI (8–12). For instance, a recreational runner has been defined as someone who runs a minimum of 3 times per week and totals 16 kilometers of running during the week (11). Comparatively, competitive runners run more frequently, and accumulate more than 100 km within a week (8, 10). The difference in exposure between different types of runners may contribute to injury susceptibility.

Running biomechanics also influence injury risk and performance. For instance, high performance runners have a longer flight phase compared with recreational runners due to faster velocity, and larger propulsive force during terminal stance (13, 14). Moreover, a lower step rate increases the risk of bone stress injury in collegiate cross-country runners (15), which may differ between different types of runners. Furthermore, lower limb position at foot contact has been investigated for its influence on injury and performance (16). A more perpendicular shank relative to the ground and closer contacts to the body's center of mass allows for greater running economy by decreasing the braking force at ground contact (16–18). A higher braking force decreases running performance and also increases risk of RRI (16, 18).

Additionally, other factors such as lower limb stiffness, ground reaction forces (GRF), and propulsion forces differ between runners and contribute to RRI and performance indicators (e.g., running speed and efficiency) (16, 17). The summed action of the ankle, knee, and hip extensors [i.e., total support moment (TSM)] contribute to propulsion during running (19). Moreover, the ankle plantarflexors contribute the largest component of positive work compared with the knee and hip extensors (20). However, these findings were in controls at relatively slower speeds, and it is unclear how joint work distribution differs between groups running at more typical training paces. Deficiency in the ankle plantarflexor moment and reliance on the knee and hip extensors in recreational runners may contribute to performance deficits and slower self-selected speeds when participating in long distance running (20). Comparisons of recreational and competitive runners elucidates optimal biomechanical strategies for performance given the known differences in self-selected running speeds.

The purpose of this study was to compare running biomechanics that have previously been linked to RRI and performance between recreational and collegiate runners. We hypothesized that collegiate runners would have more perpendicular shank angles and larger GRFs than their recreational counterparts. We also hypothesized that all lower extremity extensor moments would be higher in the collegiate group with the proportion of ankle moment as a percent of the TSM also higher in the collegiate group. A secondary purpose was to evaluate the association between GRFs and shank angle during running in recreational and collegiate runners. We hypothesized that a larger shank angle would be associated with a larger braking force in both groups.

## Materials and methods

Data used for this study were collected as part a larger study examining the association between running kinetics and femoral cartilage characteristics (21). All methods were approved by the university's institutional review board and participants provided informed written consent, before a single data collection session that lasted approximately 2-hours.

### Participants

Twenty-seven recreational and 35 collegiate runners were recruited from the university cross country team, student population, and local running groups. The criterion for being defined as a collegiate runner was determined to be currently running or running in the preceding year for an intercollegiate team (22). A recreational runner was considered to be a person running up to 3 times a week for a minimum of 10 miles (16 km) within that week (11, 21). All participants were between the ages of 18 and 35 and required to be free from lower body injuries for 6 months before participation. Further exclusion from participation included a history of lower body intra articular injections, surgery, and a body mass index (BMI) greater than 25.0 kg/m^2^.

### Running biomechanics

All participants were instructed to wear compression shorts for bottoms and either a tank top or sports bra for women and a compression shirt or shirtless for men. Standardized footwear (Nike Pegasus 32, Beaverton, OR) were provided by the laboratory to mitigate the influence of footwear being a confounding variable on running kinetics. Single retroreflective markers were placed on the posterior superior iliac spine, iliac crest, anterior superior iliac crest, greater trochanter, medial and lateral femoral epicondyles, medial and lateral malleoli, heel counter, and 1st and 5th metatarsals, while rigid clusters of 4 markers were placed on the thigh, shank, and foot. All markers were placed solely on the dominant limb. Three-dimensional running biomechanics were collected using a 20-m runway equipped with a 9-camera motion capture system recording at 240 Hz (Qualisys, Gothenburg, Sweden) and a force plate recording at 2,400 Hz (Advanced Mechanical Technology, Inc, Watertown, MA) positioned in the middle of the runway.

Upon completing a self-selected 5-min running warm up on a treadmill, participants completed 5 overground familiarization trials. Familiarization was used to ensure that participants could strike the force plate without noticeably altering their stride, and that a consistent running pace was achieved. Self-selected running speed was monitored with infrared timing gates (model TF100; TracTronix, Belton, MO) 2-m apart. Participants completed 5 trials with their dominant limb contacting the force plate and within ±5% of the self-selected speed obtained from familiarization trials.

### Data reduction

Visual 3D (C-Motion, Inc, Germantown, MD) was used for model construction. Marker trajectories and GRFs were low pass filtered at 20 Hz (23). One fourth of the intertrochanteric distance was used to estimate the hip joint center. The midpoints between the femoral epicondyles and lateral and medial malleoli were used to determine centers for the knee and ankle, respectively. Ground contact and toe-off were identified when the vertical GRF exceeded and fell below 20N, respectively, and used to determine stance phase. The strike angle was calculated as the absolute angle of a modified virtual foot segment relative to the global coordinate system to describe the footstrike pattern of the sample (24). The long axis of the foot was offset from the heel counter and distal foot markers so that the virtual foot segment was parallel to the floor during the calibration trial (24). A rearfoot strike was categorized as >8°, a midfoot strike was categorized as between −1.6° and 8°, and a forefoot strike was categorized as <−1.6° (25). The shank angle was determined relative to the global coordinate system where 0° was considered perpendicular to the ground and was extracted at the time of ground contact.

The vertical loading rate was defined as the peak derivative during the first 13% of the stance phase (22, 26), and normalized to body weight. This method allowed for comparison of loading rates between runners with various footstrike patterns who do not have an impact peak, which is common in runners with forefoot strike pattern. The peak posterior and anterior GRFs were extracted from the first and second halves of the stance phases, respectively. Impulse from the vertical and anterior-posterior GRF components was extracted using trapezoidal integration. The positive to negative ratio was determined by dividing the positive impulse by the negative impulse. Vertical loading rate and peak GRF and impulse characteristics were normalized to body weight.

**Figure 1:**
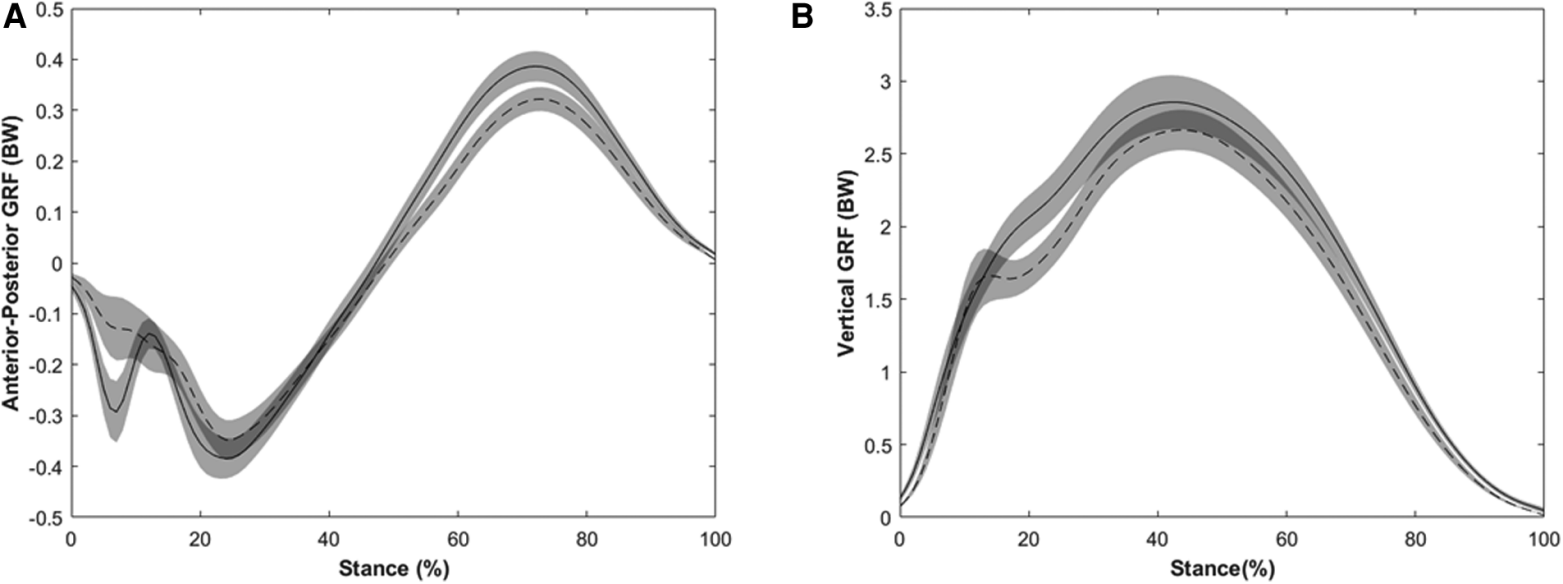
Ensemble average and 95% confidence interval for (**A**) vertical ground reaction force and (**B**) anterior-posterior ground reaction force. Solid indicates collegiate and dashed indicates recreational runners. Collegiate runners had a larger vertical impulse, propulsive force, negative impulse, positive impulse compared with recreational runners. However, recreational runners had a larger vertical LR and shank angle compared with collegiate runners.

Inverse dynamics procedures were used to derive internal hip, knee, and ankle joint moments resolved in the proximal segment coordinate system. The TSM peak values and total contributions were expressed as percentages taken at the time of peak TSM. TSM is calculated using the algebraic sum of the sagittal plane hip, knee, and ankle extensor moments (19). All extensor moments were reported as normalized to participants' height and body weight, and as percentage of TSM. Extensor moments and GRFs were time-normalized to 101 data points and plotted as ensemble average with 95% confidence interval for visualization purposes (Figure 1).

### Statistical analysis

All statistical analyses were completed using SPSS version 28.0 (IBM Corp., Armonk NY). Box plots were used to assess for outliers (>1.5× Interquartile Range) and extreme outliers (3× Interquartile Range) and normality was determined using the Shapiro-Wilk test separately for each group. The proportion of males and females, and proportion of rearfoot/midfoot/forefoot strikers were compared between groups using *χ*^2^ tests, and all other demographic characteristics were compared using independent samples *t*-tests. Ground reaction force characteristics and extensor moments were compared between groups using separate one-way multivariate analyses of variance, and *post hoc* comparisons were conducted using independent samples *t*-tests with Bonferroni correction (Family-wise *α* = 0.05). To further understand joint-level contributions to the TSM and propulsion between groups, we also compared extensor moments adjusted for self-selected speed as a co-variate. Pearson correlation was used to assess the relationship between shank angle and GRF characteristics. Correlation coefficients (*r*) were interpreted as poor (<0.3), fair (0.3–0.5), moderately strong (0.6–0.8) and very strong (>0.8) (27, 28).

## Results

Descriptive statistics of participants demographic information can be found in Table 1. The recreational runners were older than the collegiate runners. The collegiate group reported greater amount of running and ran at a greater self-selected running speed compared with the recreational group. The collegiate group also had a greater proportion of midfoot and smaller proportion of rearfoot strike runners compared with the recreational group.

**Table 1:** Participant characteristics (mean ± SD).

Demographic	Recreational (*n* = 27)	Collegiate (*n* = 35)	*P*-value
Sex (*n*)	6 female, 21 male	10 female, 25 male	0.359
Age (years)	23.6 ± 3.2	20.1 ± 1.5	<0.001
Height (m)	1.76 ± .09	1.74 ± .09	0.511
Mass (kg)	68.1 ± 9.1	61.7 ± 8.2	0.444
BMI (kg/m^2^)	22.5 ± 1.6	20.4 ± 1.9	0.228
Running amount (km)	22.0 ± 9.8	84.7 ± 15.6	0.001
Speed (m/s)	3.5 ± .46	4.1 ± .33	0.034
Footstrike pattern (*n*)			
Rearfoot	21	13	<0.001
Midfoot	2	14	
Forefoot	4	8	

BMI, Body Mass Index.

### Ground reaction force comparison

One-way multivariate analysis of variance revealed that there was a significant difference in GRF and shank outcomes between groups [Pillai's trace = 0.739, *F*(51,10) = 14.415, *p* < 0.001, Figure 1]. Post hoc comparisons demonstrated recreational runners had a larger vertical loading rate, and shank angle compared with the collegiate runners who demonstrated greater vertical impulse, negative impulse, positive impulse, and propulsive force (Table 2).

**Table 2:** Comparison of running outcomes [mean (95% confidence interval)].

	Recreational (*n* = 27)	Collegiate (*n* = 35)	*P*
Vertical GRF (BW)	2.74 (2.60, 2.89)	2.84 (2.71, 2.96)	0.324
Vertical LR (BW/s)*	194.5 (166.1, 222.9)	111.5 (86.6, 136.4)	<0.001
Vertical impulse (BW·s)*	.233 (.209, .256)	.349 (.329, .370)	<0.001
Propulsive force (BW)*	.333 (.307, .360)	.390 (.367, .413)	0.002
Braking force (BW)	−.405 (−.453, −.357)	−.443 (−.485, −.401)	0.239
Negative impulse (BW·s)*	−.013 (−.015, −.011)	−.022 (−.024, −.026)	<0.001
Positive impulse (BW·s)*	.014 (.012, .016)	.024 (.022, .026)	<0.001
Pos/Neg ratio	1.05 (.859, 1.24)	1.21 (1.04, 1.38)	0.213
Shank angle (°)*	6.80 (5.48, 8.11)	2.09 (.934, 3.25)	<0.001

BW, Body Weight; LR, Loading Rate.

* The mean difference is significant at the 0.05 level.

### Joint moment comparison

**Figure 2:**
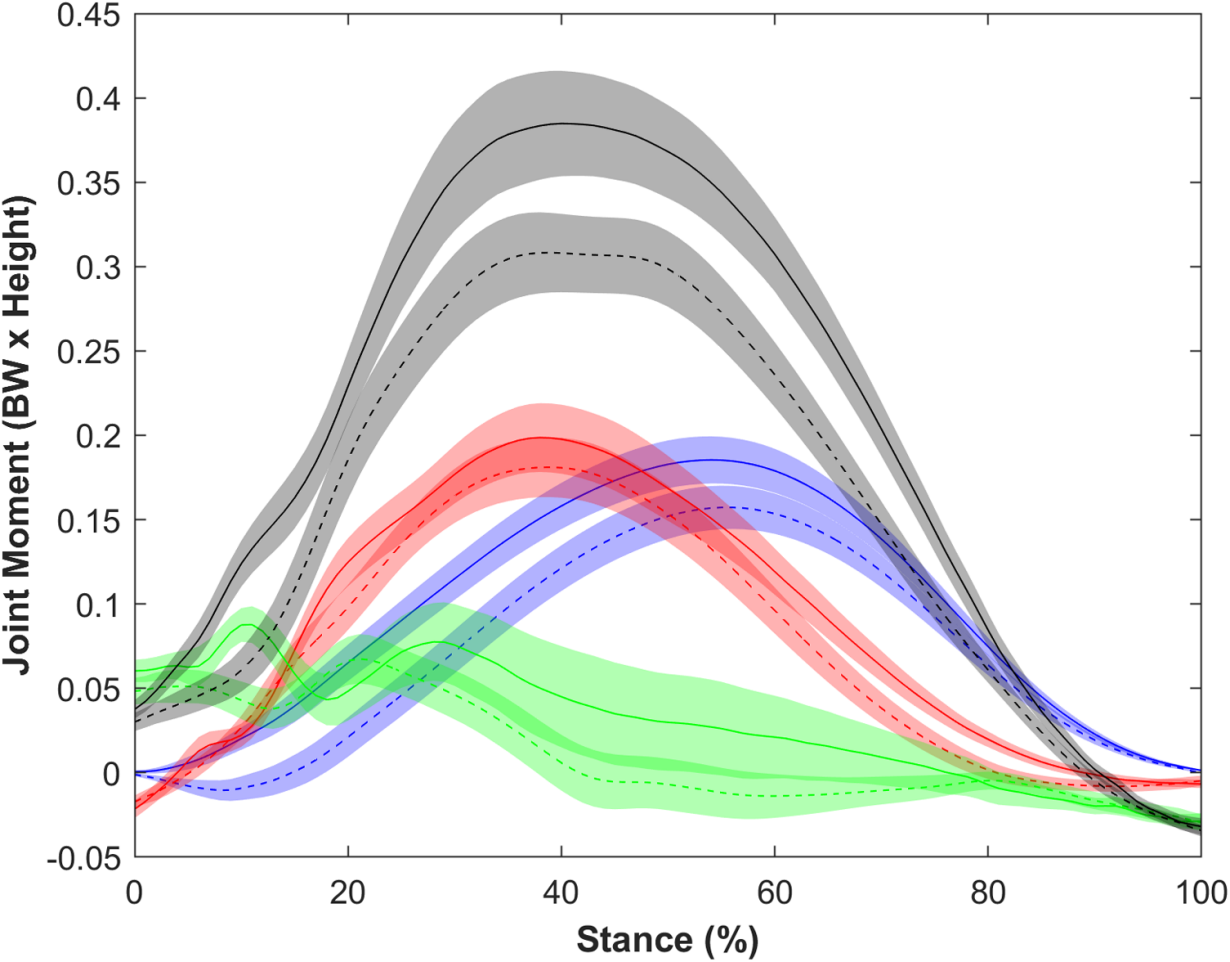
Ensemble average and 95% confidence interval for all extensor moments. Black indicates total support moment, green indicates hip extensor moment, red indicates knee extensor moment, and blue indicates plantarflexor moment. Solid indicates collegiate runners, and dashed indicates recreational runners. All joint moments were higher in collegiate compared with recreational runners.

**Figure 3:**
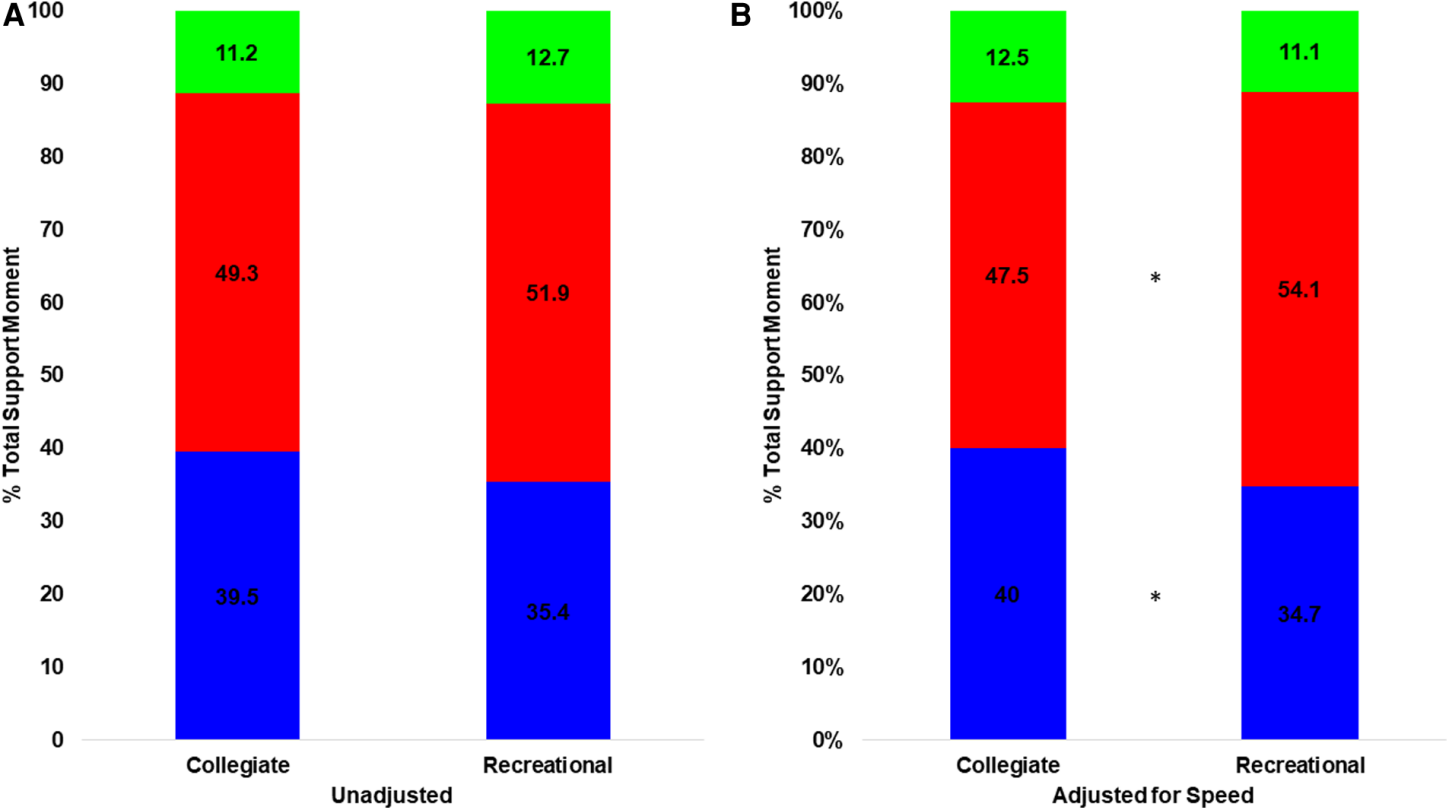
Joint moment distribution when (**A**) unadjusted and (**B**) adjusted for speed (blue is plantarflexor moment, red is knee extensor moment, green is hip extensor moment). *Indicates significant difference between groups. The plantarflexor and knee extensor moments differed between groups only when adjusted for speed.

When unadjusted for speed, normalized extensor moments differed between groups [Pillai's trace = 0.204, *F*(57,4) = 3.648, *p* = 0.01, Figures 2, 3]. Post hoc comparisons showed the collegiate group had a greater TSM (*p* = 0.002) and plantarflexor moment (*p* = 0.029) compared with recreational runners but no difference in the knee extensor moment (*p* = 0.984) and hip extensor moment (*p* = 0.064) (Table 4).

When adjusted for speed, the normalized extensor moments differed between groups [Pillai's trace = 0.342, *F*(58,4) = 7.5421, *p* < 0.001, Figure 3]. Post hoc comparisons between the recreational and collegiate group indicated that the TSM (*p* < 0.001), plantar flexor moment (*p* = 0.004), knee extensor moment (*p* = 0.033), and hip extensor moment (*p* = 0.040) were greater in collegiate runners (Table 4).

When unadjusted for speed, the individual joint contributions to the TSM did not differ between groups [Pillai's trace = 0.073, *F*(60,2) = 2.349, *p* = 0.104].

When adjusted for speed, the contributions to TSM differed between groups [Pillai's trace = 0.106, *F*(59,2) = 3.503, *p* = 0.037], and the collegiate group had a greater plantar flexor moment (*p* = 0.037) and lower knee extensor moment (*p* = 0.018) compared with the recreational group (Table 4).

### Correlational analyses

**Figure 4:**
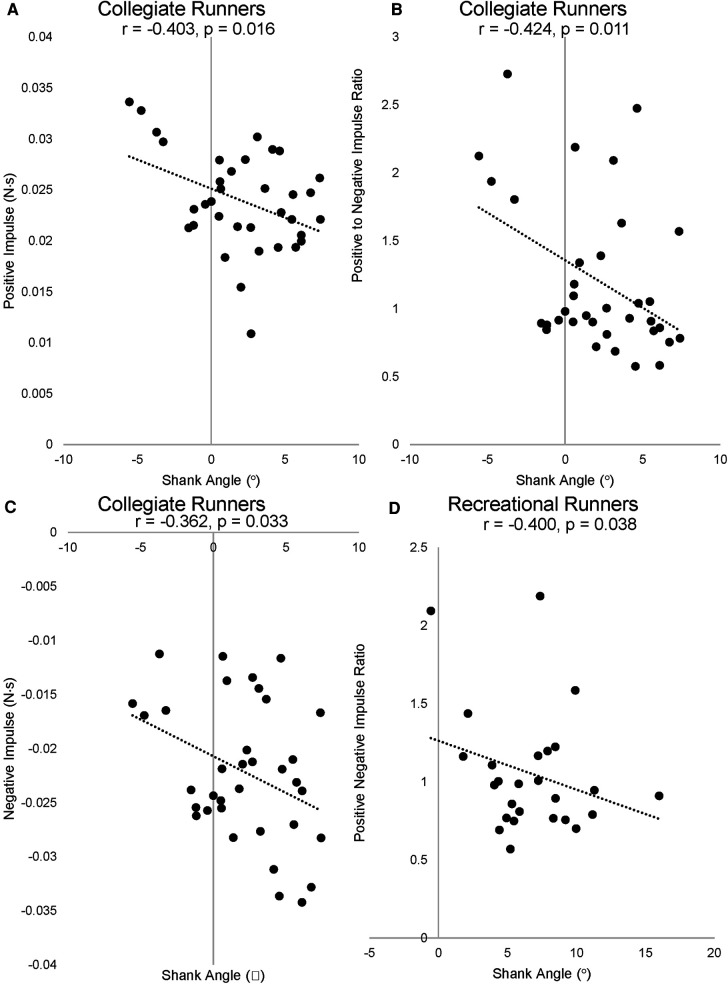
Scatterplots for significant associations between (**A**) shank angle and positive impulse in collegiate runners, (**B**) shank angle and positive to negative impulse ratio in collegiate runners, (**C**) shank angle and negative impulse in collegiate runners, and (**D**) positive to negative impulse ratio in recreational runners.

Significant and fair correlations (Figure 4, Table 3) were found between a less perpendicular shank angle relative to the ground and greater positive impulse (*r* = −0.403, *p* = 0.016), a smaller negative impulse (*r* = −0.362, *p* = 0.033), and greater positive-to-negative impulse ratio (*r* = −0.424, *p* = 0.011) in the collegiate group. A greater positive-to-negative impulse ratio was associated with a less perpendicular shank angle relative to the ground (*r* = −0.400, *p* = 0.038) in the recreational group. No other significant correlations were identified between shank angle and GRF characteristics in either group (all *p* > 0.05, Table 3).

**Table 3:** Correlations (*r*) between shank angle at ground contact and ground reaction force characteristics (*p < 0.05).

	Peak vertical GRF	Vertical LR	Vertical impulse	Propulsive force	Braking force	Negative impulse	Positive impulse	Positive to negative work ratio
Recreational runner shank angle	0.130	0.146	−0.153	0.043	−0.333	−0.124	−0.313	−0.400*
Collegiate runner shank angle	−0.139	0.234	−0.008	−0.256	−0.283	−0.362*	−0.403*	−0.424*

GRF, Ground Reaction Force; LR, Loading Rate.

* *p* < 0.05.

**Table 4:** Mean (95% confidence interval) of TSM, plantar flexor moment, knee extensor moment, and hip extensor moment controlled for body weight and height; and the plantar flexor moment, knee extensor moment, and hip extensor moment as a percentage of TSM for collegiate and recreational groups adjusted and unadjusted for speed.

	Not adjusted for speed		Adjusted for speed	
	Rec (*n* = 27)	Collegiate (*n* = 35)	Rec (*n* = 27)	Collegiate (*n* = 35)
Total support moment (BW × Ht)	0.319 (0.299, 0.339)*	0.393 (0.375, 0.411)	0.329 (0.305, 0.352)*	0.385 (0.365, 0.406)
Plantar flexor moment (BW × Ht)	0.125 (0.109, 0.141)*	0.156 (0.142, 0.170)	0.125 (0.107, 0.144)*	0.156 (0.140, 0.172)
Knee extensor moment (BW × Ht)	0.176 (0.162, 0.189)*	0.196 (0.183, 0.208)	0.187 (0.172, 0.202)*	0.187 (0.173, 0.200)
Hip extensor moment (BW × Ht)	0.019 (0.002, 0.035)*	0.041 (0.027, 0.056)	0.016 (−0.003, 0.036)*	0.043 (0.026, 0.060)
Plantar flexor moment (%TSM)	0.354 (0.325, 0.382)	0.395 (0.370, 0.421)	0.347 (0.314, 0.381)*	0.400 (0.371, 0.429)
Knee extensor moment (%TSM)	0.519 (0.486, 0.552)	0.493 (0.464, 0.522)	0.541 (0.504, 0.578)*	0.475 (0.443, 0.507)
Hip extensor moment (%TSM)	0.127 (0.096, 0.159)	0.112 (0.084, 0.140)	0.111 (0.075, 0.147)	0.125 (0.093, 0.156)

* Different from collegiate group; *p* < 0.05.

## Discussion

**Figure 5:**
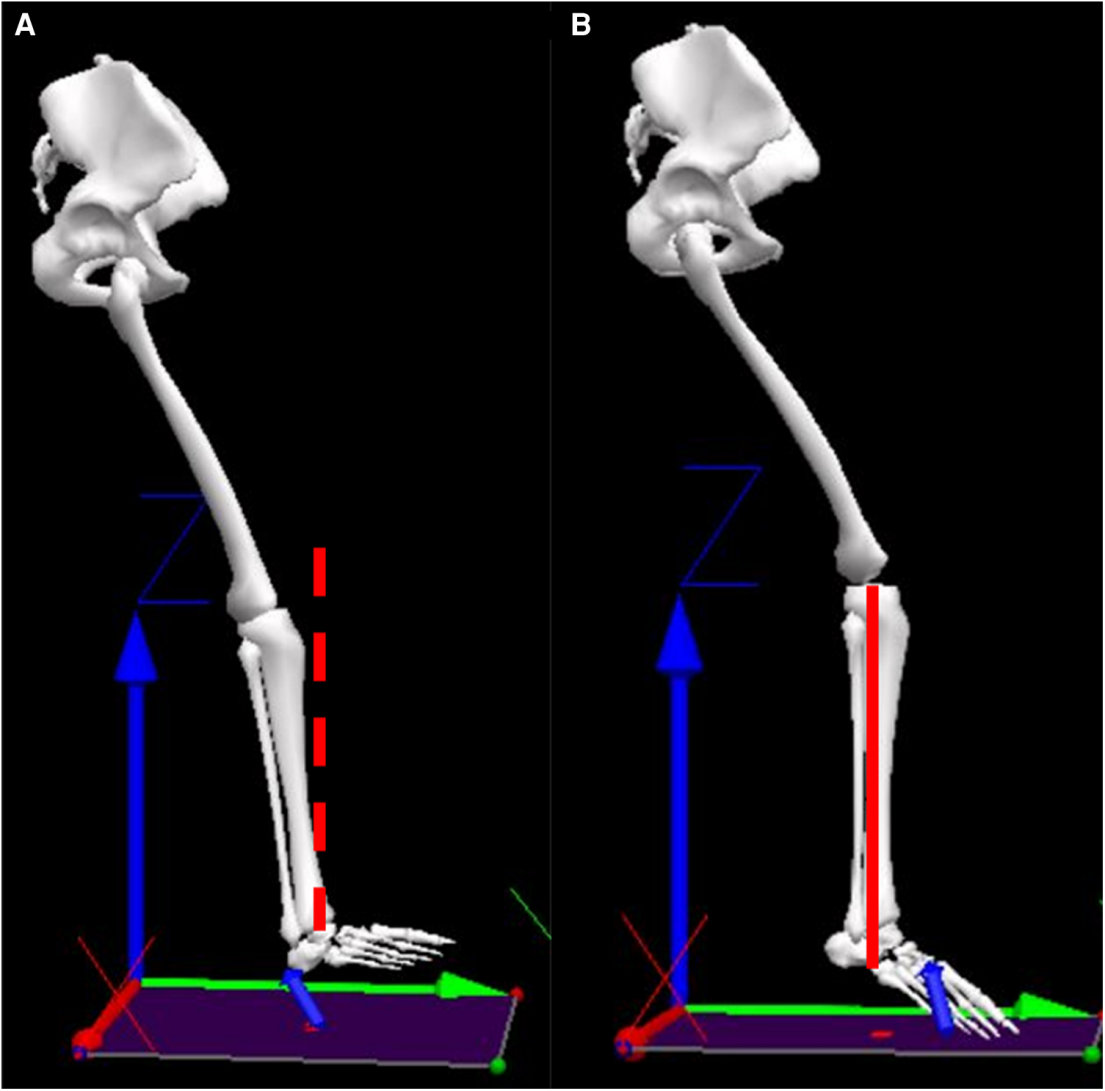
Representative figure of (**A**) recreational runner and (**B**) collegiate runner where the shank angle is larger versus upright, respectively.

The primary purpose of this study was to compare injury- and performance- related running mechanics between recreational and collegiate runners. We further examined the association between ground contact and GRF characteristics within each group. The main findings indicated that collegiate runners used a larger proportion of the ankle extensors, but smaller proportion of the knee extensors compared with recreational runners. Moreover, collegiate runners had larger propulsive features (e.g., anterior force, vertical impulse) compared with recreational runners, and a more vertically oriented shank at ground contact (Figure 5). A more vertically oriented shank was associated with a larger propulsive impulse and smaller braking impulse.

We hypothesized that the joint moment distribution would differ between collegiate and recreational runners. Adjusting for speed, we found that the collegiate runners used a larger proportion of ankle extensor moment compared with recreational runners. The ankle plantar flexors are a large contributor to propulsion during running, and comprise the largest proportion of lower extremity joint work compared with the knee or hip extensors (20). Moreover, there is a distal to proximal shift in joint work during gait as speed increases from walking to sprinting (20, 29). As such, these findings suggest that collegiate runners use a more efficient strategy when speed is controlled that uses the ankle plantar flexors for propulsion. Moreover, the ability to utilize a larger proportion of the ankle plantar flexors when adjusting for speed likely increases their maximal running speed capacity relative to recreational runners and allow them to run at faster sub-maximal speeds for longer durations. Ankle power generation increases with faster running speeds and contributes to propulsion in recreational runners (14). Therefore, improving ankle plantar flexor function to reduce reliance on the hip and knee extensors during running may be a strategy to improve running performance in recreational runners and achieve similar self-selected speeds as collegiate runners during prolonged running.

The recreational runners had a larger knee extensor moment expressed as a percentage of TSM compared with collegiate runners when comparisons were adjusted for speed. A larger internal knee extensor moment contributes to greater patellofemoral contact stress (30, 31). As such, recreational runners utilize a running strategy that contributes to a disproportionate load on the patellofemoral joint that elevates risk for anterior knee pain (30, 32). Recreational runners may reduce their risk of injury from increasing the proportion of contribution from the ankle and hip joint extensor moments to the TSM. This contributes to reductions in knee extensor moment needed during running and decrease load to the patellofemoral joint. Conversely, collegiate runners may experience foot/ankle pathologies due to additional reliance on the ankle plantar flexors (33–35).

The collegiate runners in our sample had higher extensor moments but similar distribution of moments at every joint compared with recreational runners when comparisons were unadjusted for speed. These findings were expected and likely contributed to the overall difference in anterior force and self-selected running speed. Collegiate runners may have greater muscle capacity for joint extension from completing a larger training volume or supplementary resistance training (12, 36). These findings were supported by GRF comparisons, and collegiate runners who had greater propulsive force, and positive impulse compared with recreational runners. Supplementary forms of strength training with endurance training improves running economy, power, reactive strength, and running performance in collegiate runners (36). Therefore, improvements in running performance could be achieved in recreational runners by participating in supplementary resistance training similar to collegiate runners. Supplementary training may increase their muscle capacity at all joints for propulsive forces and positive impulse to the values that we observed in the collegiate group. Future research may aim to investigate if the additional of strength training to recreational runners could improve running speed.

We also found that recreational runners had higher vertical loading rates compared with collegiate runners when normalized to body weight. The vertical loading rate has been retrospectively (37), and prospectively (38), linked to tibial stress fracture (39, 40). However, these studies have largely been limited to recreational runners. In conjunction with our data, recreational runners may be at greater risk for stress fracture from elevated vertical loading rates. The vertical loading rate is strongly influenced by speed and foot strike pattern (41). As running speed increases, the vertical loading rate increases (41). Faster running speed also contribute to shift from rearfoot to a more anterior foot strike pattern for some runners (41). A more anterior foot strike pattern has been associated with reduced vertical loading rate compared with rearfoot contact at the same running speed (41). Collegiate runners in our sample ran faster and had a lower proportion of rearfoot strike runners. As such, faster speed and higher loading rates may have been mitigated by running with a more forefoot pattern (41). Collectively, these data suggest that collegiate and recreational runners have different biomechanics that contribute to unique injury patterns, and prospective data are needed to evaluate these hypotheses.

We also examined the association between shank kinematics at ground contact and GRF characteristics and found that a more vertically oriented shank was associated with less negative impulse and more positive impulse. The braking/posterior force has been prospectively linked to running-related injury (42), and a positive impulse is linked to faster running speeds (43). A prospective study on female recreational runners found that injury incidence was 8 times higher for runners with peak braking forces 0.27× body weight compared with 0.23× body weight (42). Moreover, a retrospective study found limbs with previous tibial stress fractures to demonstrate greater peak braking force values (44). Bone is stronger under compressive forces rather than shear force (45). Increased braking force could contribute to shear forces and greater bone-stress injury risk (44, 46). Furthermore, faster runners can apply 1.26 times greater average force per body weight in less time on the ground compared with slower runners, achieving 1.8 times faster top speeds (43). Elite runners have greater vertical forces 0.16 BW higher, and more vertical shank angle at ground contact compared with sub-elite runners (17). Therefore, our findings suggest that manipulating shank position presents a viable target for gait modification that may influence both injury risk and running performance. A more vertical shank angle at ground contact has been associated with better running performance and minimizes horizontal braking, which has been associated with better running economy and performance in distance runners (16). Furthermore, between laps one and two of an 800 m, the faster of the two laps presented a more vertical shank angle at ground contact in female distance runners at the world athletics championships (47). Our findings of a more vertically oriented shank in the collegiate group compared with recreational therefore, could be a biomechanical contributor to their faster running speeds. However, we note that our sample included some collegiate runners whose shank angles were slightly negative (i.e., leaned forward) rather than perpendicular, which may not be ideal. Moreover, correlation coefficients were only fair, and left a large portion of unexplained variance in GRF characteristics. Therefore, future studies should evaluate optimal shank angle during ground contact for both performance and injury prevention purposes.

Results of this study should be interpreted in the context of its limitations. Firstly, the cross-sectional study design negates the ability to evaluate if running mechanics are a result of different training magnitudes and behaviours of recreational and collegiate runners. Nonetheless, we still identified differences in running biomechanics that may contribute to the difference in ability of recreational and collegiate runners. Prospective data are needed to evaluate if running patterns change as a result of habitual training and can be manipulated via interventions (e.g., supplemental resistance training, biofeedback etc.). Secondly, we only examined self-selected running speeds to approximate habitual running. Our comparisons that statistically adjusted for running speed found differences in ankle and knee extensor contributions to the TSM that may influence propulsion. However, comparisons at matched speeds may further elucidate unique locomotive strategies that differentiate collegiate and recreational runners.

## Conclusion

Collegiate runners compared with recreational runners demonstrated greater propulsion and a more vertically oriented shank that was associated with higher propulsive impulse and lower braking impulse. As such, runners could reduce braking forces that have previously shown association to tibial stress fractures by running with a more vertically oriented shank angle (42, 44, 46). A more vertical shank could also contribute to faster running through greater propulsion and less braking. Therefore, future studies may investigate the influence of altering shank angles on running performance and injury risk. Collegiate runners also used a greater proportion of the ankle extensors and smaller proportion of knee extensors than recreational runners when adjusted for speed. A greater contribution from the ankle joint compared with knee and hip at slower speeds may provide a more efficient propulsion strategy that allows for a larger capacity to increase running speed. Therefore, recreational runners may be able to achieve faster running speeds through greater proportional use of their ankle plantar flexors during propulsion.

## Data Availability

The raw data supporting the conclusions of this article will be made available by the authors, without undue reservation.
